# Benefits of biphasic calcium phosphate hybrid scaffold-driven osteogenic differentiation of mesenchymal stem cells through upregulated leptin receptor expression

**DOI:** 10.1186/s13018-015-0236-2

**Published:** 2015-07-16

**Authors:** Chi-Chien Niu, Song-Shu Lin, Wen-Jer Chen, Shih-Jung Liu, Lih-Huei Chen, Chuen-Yung Yang, Chao-Jan Wang, Li-Jen Yuan, Po-Han Chen, Hsiao-Yang Cheng

**Affiliations:** Department of Orthopaedics, Chang Gung Memorial Hospital, No. 5, Fu-Hsing Street 333, Kweishan, Taoyuan, Taiwan; College of Medicine, Chang Gung University, Taoyuan, Taiwan; Department of Mechanical Engineering, Chang Gung University, Taoyuan, Taiwan; Department of Radiology, Chang Gung Memorial Hospital, Taoyuan, Taiwan

**Keywords:** Coralline HA, BCP, MSCs, Osteogenesis, Leptin receptor

## Abstract

**Background:**

The use of mesenchymal stem cells (MSCs) and coralline hydroxyapatite (HA) or biphasic calcium phosphate (BCP) as a bone substitute for posterolateral spinal fusion has been reported. However, the genes and molecular signals by which MSCs interact with their surrounding environment require further elucidation.

**Methods:**

MSCs were harvested from bone grafting patients and identified by flow cytometry. A composite scaffold was developed using poly(lactide-*co*-glycolide) (PLGA) copolymer, coralline HA, BCP, and collagen as a carrier matrix for MSCs. The gene expression profiles of MSCs cultured in the scaffolds were measured by microarrays. The alkaline phosphatase (ALP) activity of the MSCs was assessed, and the expression of osteogenic genes and proteins was determined by quantitative polymerase chain reaction (Q-PCR) and Western blotting. Furthermore, we cultured rabbit MSCs in BCP or coralline HA hybrid scaffolds and transplanted these mixtures into rabbits for spinal fusion. We investigated the differences between BCP and coralline HA hybrid scaffolds by dual-energy X-ray absorptiometry (DEXA) and computed tomography (CT).

**Results:**

Tested in vitro, the cells were negative for hematopoietic cell markers and positive for MSC markers. There was higher expression of 80 genes and lower of 101 genes of MSCs cultured in BCP hybrid scaffolds. Some of these genes have been shown to play a role in osteogenesis of MSCs. In addition, MSCs cultured in BCP hybrid scaffolds produced more messenger RNA (mRNA) for osteopontin, osteocalcin, Runx2, and leptin receptor (leptin-R) than those cultured in coralline HA hybrid scaffolds. Western blotting showed more Runx2 and leptin-R protein expression in BCP hybrid scaffolds. For in vivo results, 3D reconstructed CT images showed continuous bone bridges and fusion mass incorporated with the transverse processes. Bone mineral content (BMC) values were higher in the BCP hybrid scaffold group than in the coralline HA hybrid scaffold group.

**Conclusions:**

The BCP hybrid scaffold for osteogenesis of MSCs is better than the coralline HA hybrid scaffold by upregulating expression of leptin-R. This was consistent with in vivo data, which indicated that BCP hybrid scaffolds induced more bone formation in a spinal fusion model.

## Introduction

Autogenous bone is the most effective graft material for the repair of bone defects or for spinal fusion; however, donor site morbidity is a major limitation on its clinical use [1]. To avoid this limitation, one tissue engineering approach would be to combine cells capable of osteogenic activity with an appropriate scaffolding material to stimulate bone regeneration and repair. The use of calcium phosphate ceramics as substitutes for bone grafts is steadily increasing due to ongoing improvement of the materials. The most widely used calcium phosphate ceramics are porous hydroxyapatite (HA), phosphate (β-TCP), and mixtures of these two that are known as biphasic calcium phosphate (BCP) ceramics [2–4].

Mesenchymal stem cells (MSCs) have the potential to differentiate into lineages of various mesenchymal tissues, including bone, cartilage, fat, tendon, and muscle. Many surface antigens are often expressed on human MSCs, such as CD146, CD106, CD105, CD90, Stro-1, and α-SMA [5, 6]. MSCs have great appeal for tissue engineering and therapeutic applications. MSCs combined with HA [7] or BCP [8] ceramics have been shown to induce bone formation. In addition, the use of MSCs on HA [9] or BCP [10] as a bone substitute for posterolateral spinal fusion also has been reported. However, the molecular and biochemical signals by which MSCs interact with their surrounding environment require further elucidation to develop improved scaffolding materials.

In the process of biological developmental, osteoblasts differentiate from mesenchymal progenitors, going through distinct developmental stages that are regulated by various developmental signals and signaling pathways. These pathways include BMP signaling [11], Hedgehog signaling [12], Notch signaling [13], Wnt signaling [14], FGF signaling [15], and leptin–leptin-R signaling [16]. Leptin is a 16-kDa peptide hormone product of the ob (Lep) gene that is secreted by white adipose tissue [17], human osteoblasts [18], and MSCs [19]. Leptin-R is expressed on the surface of human bone marrow MSCs [20]. Leptin has a significant effect of promoting osteogenesis and inhibiting adipogenesis in MSCs. Runx2 messenger RNA (mRNA) expression is upregulated by leptin during osteogenesis in MSCs, while leptin reduced PPARγ2 mRNA expression during adipogenesis in MSCs [21, 22]. Combined treatment with leptin and rhBMP-2 has a synergistic osteoinductive activity in nude mice [23]. In addition, leptin inhibits osteoclast generation [24]. Leptin increased osteoprotegerin (OPG) mRNA and protein expression in MSCs, and the inhibitory effect may be mediated by the RANKL/RANK/OPG system [24]. Leptin activity in MSCs from osteoporotic women appears hampered, suggesting that inadequate leptin activity contributes to excessive lipid accumulation in the bone marrow [21, 22].

Previous studies have employed various molecular methods to analyze gene expression in cells and tissues in contact with biomaterials and their dissolution products [25, 26]. In the present study, we used alkaline phosphatase (ALP) activity, quantitative polymerase chain reaction (Q-PCR), and Western blotting to analyze gene and protein expression during osteogenesis of MSCs cultured in different combinations of osteoconductive materials (HA, BCP, and collagen). By comparing the expression profile of leptin, leptin-R, ALP, osteopontin (OPN), osteocalcin (OSC), and Runx2, we first proved that calcium phosphate ceramics could stimulate the differentiation of MSCs via influencing the expression of molecules in the leptin/leptin-R/ALP/OSC signaling pathway. In addition, we cultured rabbit MSCs in BCP or HA hybrid scaffolds and transplanted these mixtures into rabbits for posterior spinal fusion. We investigated the differences between BCP and HA hybrid scaffolds by dual-energy X-ray absorptiometry (DEXA) and computed tomography (CT) examination.

## Materials and methods

The experimental protocol was approved by the human subjects Institutional Review Board and the Institutional Animal Care and Use Committee of the Chang Gung Memorial Hospital.

### Surgical procedures

MSCs were harvested from patients who underwent iliac bone grafting for spine fusion. For each patient, 10 mL of bone marrow was aspirated and collected in a sterile heparin-rinsed syringe.

### Isolation and cultivation of MSCs

Each marrow sample was washed with phosphate-buffered saline (PBS). Up to 2 × 10^8^ nucleated cells in 5 mL of PBS were loaded onto 25 mL of Percoll cushion (Pharmacia Biotech). A density gradient was used as the isolation procedure to eliminate unwanted cell types. A small percentage of cells were isolated from the density interface at 1.073 g/mL. The cells were resuspended and plated in T-75 flasks at 2 × 10^5^ cells per flask. The cells were maintained in Dulbecco’s Modified Eagle’s Medium-Low Glucose (DMEM-LG; Gibco, Grand Island, NY) containing 20 % fetal bovine serum (FBS) and antibiotics at 37 °C in a humidified atmosphere of 5 % CO_2_ and 95 % air. After 7 days of primary culturing, the non-adherent cells were removed by changing the medium. The MSCs grew as symmetric colonies and were subcultured at 10 to 14 days by treatment with 0.05 % trypsin (Gibco) and seeded into fresh flasks.

### Flow cytometric analysis of surface antigen expression

When confluent, the MSCs were passaged 1 in 3, and a sample was analyzed by flow cytometry for MSC marker expression. The cells were washed in PBS and then removed from the flask by 0.05 % trypsin (Gibco). 1 × 10^5^ cells were incubated with each mouse monoclonal primary antibody at 4 °C for 30 min. Mouse FITC-conjugated anti-CD105 antibody (1:100 dilution) and mouse FITC-conjugated anti-CD34 antibody (1:100 dilution) were purchased from Becton Dickinson (Oxford, UK). Mouse FITC-conjugated anti-αSMA antibody (1:25 dilution) was purchased from Abcam (Cambridge, UK). Mouse PE-conjugated anti-STRO-1 antibody (1:50 dilution) was purchased from Santa Cruz (CA, USA). After wash, the cells were resuspended in 500 μL wash buffer and analyzed on a BD flow cytometer (Oxford, UK).

### Construction of the PLGA–calcium phosphate–collagen carrier for MSCs

A composite system was developed using poly(lactide-*co*-glycolide) (PLGA) copolymer, coralline HA, BCP ceramics, and collagen as a carrier matrix for MSCs. PLGA (300 mg; Boehringer Ingelheim, Germany) and coralline HA ceramics (100 mg; Pro-Osteon 500R, Interpore International, Irvine, CA) or BCP (15 % HA/85 % β-TCP 100 mg; MASTERGRAFT® Granules, BioHorizons IPH Inc., USA) were loaded into molds and hot compression molded at 55 °C to form a PLGA/coralline HA or PLGA/BCP capsule graft. Roughly, 10^7^ MSCs were mixed well with 0.8 mL of type I collagen solution (Roche, Germany), 0.1 mL of 0.2 mol/L HEPES (pH 7.3), and 0.1 mL of 10× concentrated complete medium (DMEM-LG) or osteogenic medium (DMEM-LG containing 100 mmol/L ascorbate-2 phosphate, 10^−7^ M dexamethasone, 10 mmol/L β-glycerophosphate, and 20 % FBS) and stored at 4 °C. Approximately 250 μL of the MSC–collagen mixture was loaded into the cores of a PLGA/coralline HA or PLGA/BCP capsule and incubated at 37 °C for 8 h to enable collagen gel formation. The composite systems were cultured in six-well plates containing 2.5 mL of complete medium or osteogenic medium and incubated at 37 °C. The medium was changed per 72 h.

### Quantitative measurement of ALP activity

After culturing for 14 days, the culture medium was withdrawn and the MSC carrier was washed twice with 5 mL of Tyrode’s balanced salt solution. A 5-mL aliquot of ALP substrate buffer (50 mmol/L glycine, 1 mmol/L MgCl_2_, pH 10.5), containing the soluble chromogenic ALP substrate (2.5 mmol/L *p*-nitrophenyl phosphate), was added at room temperature. During incubation, ALP converts *p*-nitrophenyl phosphate into *p*-nitrophenol, which is yellow in color. Twenty minutes after substrate addition, 1 mL of the buffer was withdrawn and mixed with 1 mL of 1 N NaOH to stop the reaction. The absorbance of the mixture was read in triplicate at 405 nm on an ELISA MRX plate reader (Dynatech Labs, Chantilly, VA). Enzyme activity was expressed as nanomoles *p*-nitrophenol per minute per dish.

### RNA preparation and Q-PCR analysis

After culturing for 7 or 14 days, total RNA was extracted using a Qiagen RT kit (Qiagen, USA) according to the manufacturer’s instructions. The RNA concentration was evaluated by A260/A280 measurement. To detect leptin-R, OPN, OSC, Runx2, and glyceraldehyde 3-phosphate dehydrogenase (GAPDH) RNA transcripts, cDNA was analyzed on an ABI PRISM 7900 sequence detection system using TaqMan PCR Master Mix (Applied Biosystems, Foster City, CA). The cycle threshold (Ct) values were obtained, and the data were normalized to GAPDH expression by using the ΔΔCt method to calculate the relative mRNA level of each gene.

### Western blot analysis

After culturing for 7 or 14 days, the cells were washed with PBS and extracted using M-PER protein extraction reagent (Thermo, USA). The protein content was quantitated using a protein assay kit (Pierce Biotechnology, IL), separated by 7.5 % SDS-PAGE, and transferred onto membranes using a transfer unit (Bio-Rad, USA). After blocking, the membranes were incubated with 1000-fold diluted rabbit antibodies against leptin (R&D, MN, USA), leptin-R (R&D), Runx2 (Millipore, Temecula, CA), and β-actin (Millipore). After washing, the membranes were further incubated for 2 h with 10,000-fold goat anti-mouse IgG (Calbiochem, USA) or goat anti-rabbit IgG (Millipore) conjugated to horseradish peroxidase. The membranes were then washed and rinsed with ECL detection reagents (Millipore). The bands were photographed using ECL Hyperfilm (Amersham Pharmacia Biotech, UK), and their intensity was quantified using an image analysis system (Image-Pro plus 5.0).

### Microarray analysis

After culturing, cells were collected for studying the differential gene expression pattern using Affymetrix Human Genome U133 plus microarrays (Affymetrix, Santa Clara, CA) according to the manufacturer’s protocol. RNA preparation was performed using Qiagen’s RNeasy Mini Kit, and the integrity and purity were analyzed using Agilent Bioanalyzer and NanoDrop spectrophotometer. Raw gene expression data were normalized and analyzed with GeneChip Operating Software 1.4 (GCOS, Affymetrix). Genes that are >1.5-fold up- or downregulated between two groups were selected.

### Animal experiments and operative procedures

Six New Zealand white rabbits, each about 8 months old and weighing 3.3 to 3.8 kg, were used. Under anesthesia, a 6-cm dorsal midline incision was made to expose the bilateral paramedian fascia. The intermuscular plane between the multifidus and longissimus muscles was dissected to expose the intertransverse space between L4 and L5. The soft tissues between the L4 and L5 transverse processes were dissected, and decortication of the transverse processes was performed by using an electric burr. An MSC/PLGA/BCP/collagen graft was placed on the left side and a MSC/PLGA/coralline HA/collagen graft was placed on the right side between the transverse processes in the paraspinal bed. The fascia and skin incisions were sutured. The rabbits were killed at 10 weeks after grafting and underwent radiographic, CT, and bone mineral content (BMC) examinations.

### Computed tomography examination

At 10 weeks after grafting, three rabbits underwent high-resolution helical CT scanning at a 2.0-mm slice resolution in 1.0-mm increments. CT images were transferred to a workstation and reconstructed three-dimensionally. Multiple slices obtained in three planes were reviewed for each fusion mass.

### Dual-energy X-ray absorptiometry examination

At 10 weeks after grafting, three rabbits underwent BMC measurements by using a Hologic QDR 2000 dual-energy X-ray absorptiometry system (Hologic, Inc., Boston, MA). A self-contained X-ray source was mounted beneath an animal to provide alternating pulses at 70 and 140 kVp. An X-ray detector was then mounted above the animal, and both source and detector were moved across the animal under computer control in a serpentine pattern. The two radiation energies were generated as collimated beams and their absorption patterns allowed for the measurement of BMC in grams.

## Results

### In vitro studies

#### Flow cytometry analysis

**Fig. 1:**
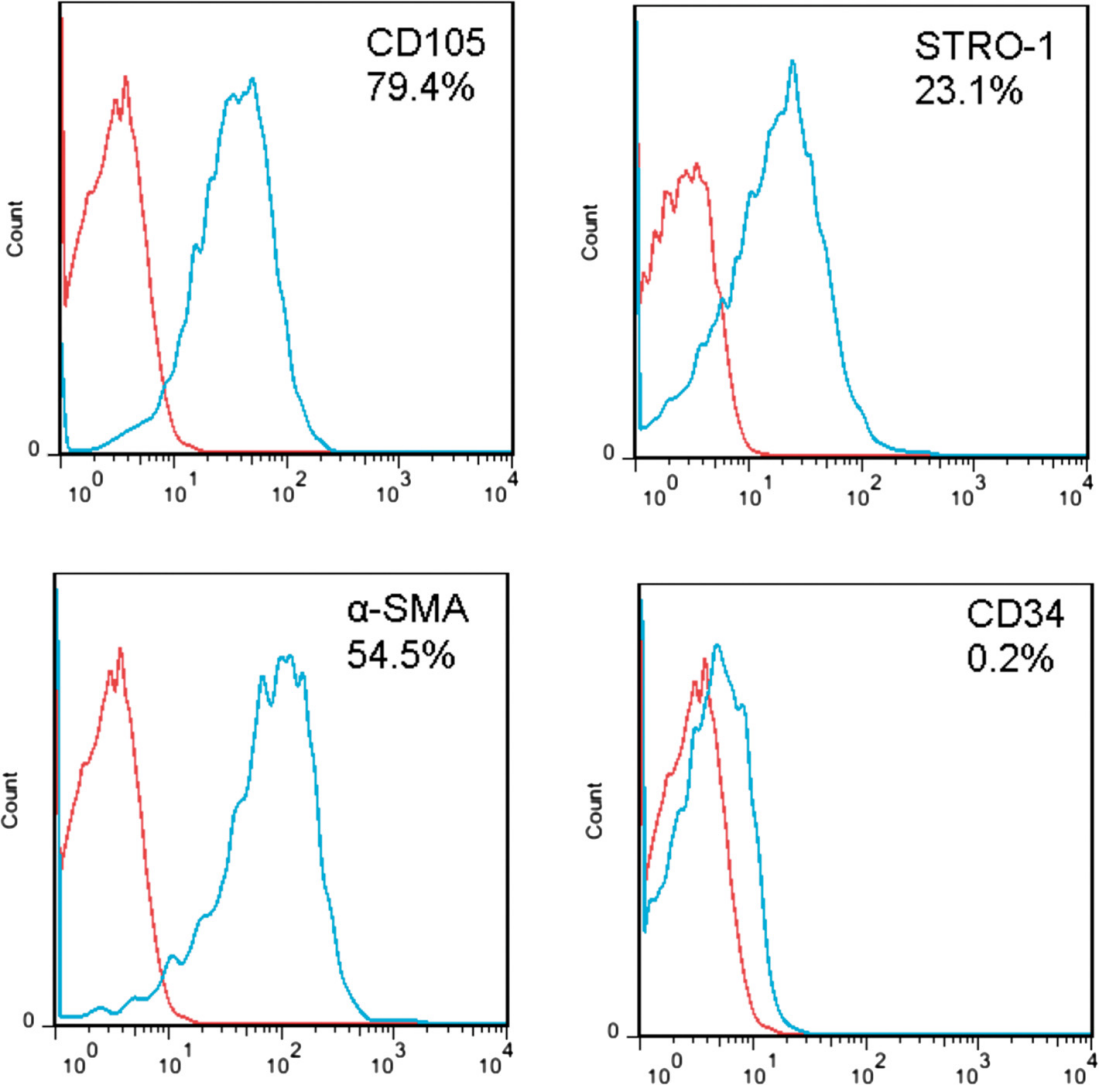
Flow cytometry analysis. The mean percentages of CD105^+^, Stro-1^+^, α-SMA^+^, and CD34^+^ cells were calculated to be 85.7 ± 5.7, 32.7 ± 4.9, 53.6 ± 1.8, and 0.21 ± 0.07 %, respectively

Primary adherent human MSCs from three donors were cultured in a control medium, and the cells were analyzed for expression of MSC markers using flow cytometry at passage 1. The percentage of cells expressing the MSC markers CD105, Stro-1, and α-SMA and the hematopoietic stem cell (HSC) marker CD34 are shown in Fig. 1. The mean percentages of CD105^+^, Stro-1^+^, α-SMA^+^, and CD34^+^ cells in the cell preparations from three patients were calculated to be 85.7 ± 5.7, 32.7 ± 4.9, 53.6 ± 1.8, and 0.21 ± 0.07 %, respectively.

#### Gene expression profiling

MSCs cultured in different scaffolds modulate the expression of several genes, leading to higher expression of 80 genes (BCP hybrid scaffold/coralline HA hybrid scaffold ratio >1.5-fold) and lower expression of 101 genes (BCP hybrid scaffold/coralline HA hybrid scaffold ratio <0.67-fold) in BCP hybrid scaffolds in 22,215 human genes. The top 12 higher and lower gene expressions of MSCs cultured in BCP hybrid scaffolds are shown in Table 1.

**Table 1:** Microarray analyses of the top 12 higher and lower gene expressions of MSCs cultured in BCP hybrid scaffolds

Gene symbol	Entrez Gene	RefSeq transcript ID	BCP hybrid scaffold/HA hybrid scaffold fold changes
(a) Higher			
LEPR	3953	NM_001003679 /// NM_001003680 /// NM_002303	4.25
MAFB	9935	NM_005461	2.99
IFI16	3428	NM_005531	2.81
FLJ10159	55084	NM_018013	2.35
MMP13	4322	NM_002427	2.27
COL21A1	81578	NM_030820	2.09
EIF4A1	1973	NM_001416	2.02
SEPP1	6414	NM_005410	1.95
APOE	348	NM_000041	1.94
RUNX2	860	NM_001015051 /// NM_001024630 /// NM_004348	1.94
PTGFR	5737	NM_000959	1.93
OLFML2B	25903	NM_015441	1.89
(b) Lower			
INHBA	3624	NM_002192	0.50
WSB1	26118	NM_015626 /// NM_134264 /// NM_134265	0.49
GOLGA8A	23015	NM_015003	0.48
STMN2	11075	NM_007029	0.48
RIS1	25907	NM_015444	0.48
PPAP2B	8613	NM_003713 /// NM_177414	0.47
PWP1	11137	NM_007062	0.46
IQGAP1	8826	NM_003870	0.43
COMP	1311	NM_000095	0.41
RGC32	28984	NM_014059	0.38
STC1	6781	NM_003155	0.36
PGK1	5230	NM_000291	0.33

#### mRNA expression of osteogenic genes and leptin-R

**Fig. 2:**
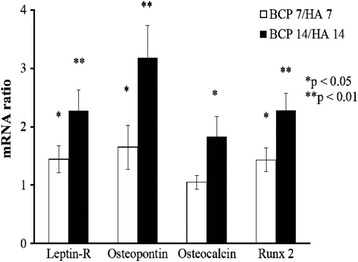
mRNA expression of osteogenic genes and leptin-R. After 7 days of culture, the mRNA levels of leptin-R (*p < 0.05, *n* = 3), osteopontin (*p < 0.05, *n* = 3), and Runx2 (*p < 0.05, *n* = 3) were significantly higher in the PLGA/BCP-group MSCs than in the PLGA/coralline-derived-HA-group MSCs. After 14 days of culture, the mRNA levels of leptin-R (**p < 0.01, *n* = 3), osteopontin (**p < 0.01, *n* = 3), osteocalcin (*p < 0.05, *n* = 3), and Runx2 (**p < 0.01, *n* = 3) were significantly higher in the PLGA/BCP-group MSCs than in the PLGA/coralline-derived-HA-group MSCs

Figure 2 presents the mRNA expression of osteogenic genes and leptin-R in the MSCs. After 7 days of culture, the mRNA levels of leptin-R (1.44 ± 0.23-fold, *p < 0.05, *n* = 3), osteopontin (1.65 ± 0.38-fold, *p < 0.05, *n* = 3), and Runx2 (1.43 ± 0.2-fold, *p < 0.05, *n* = 3) were significantly higher in the PLGA/BCP-group MSCs than in the PLGA/coralline-HA-group MSCs. After 14 days of culture, the mRNA levels of leptin-R (2.27 ± 0.36-fold, **p < 0.01, *n* = 3), osteopontin (3.18 ± 0.55-fold, **p < 0.01, *n* = 3), osteocalcin (1.83 ± 0.34-fold, *p < 0.05, *n* = 3), and Runx2 (2.28 ± 0.29-fold, **p < 0.01, *n* = 3) were significantly higher in the PLGA/BCP-group MSCs than in the PLGA/coralline-HA-group MSCs.

#### Osteogenic protein expression

**Fig. 3:**
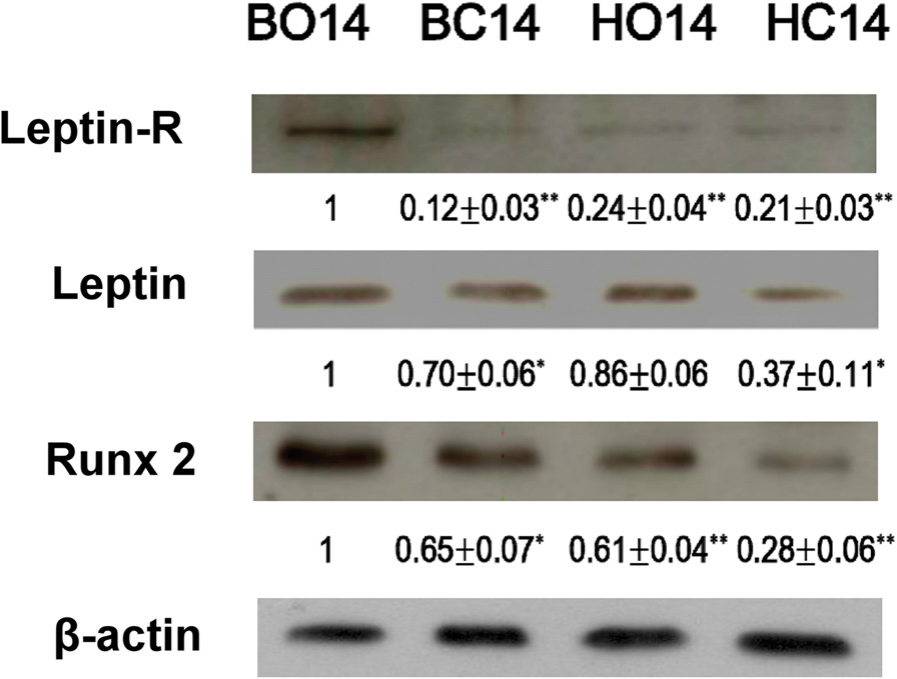
Osteogenic protein expression. Compared to a control group, the expression of the detected osteogenic proteins in the coralline-derived-HA-group MSCs and in the BCP-group MSCs was considerably enhanced. After 14 days of culture, the protein levels of leptin-R (**p < 0.01, *n* = 3), leptin (0.70 ± 0.06-fold, *p < 0.05, *n* = 3), and Runx2 (*p < 0.05, *n* = 3) were significantly higher in the BCP/osteogenic-group MSCs than in the BCP/control-group MSCs. Additionally, the protein levels of leptin-R (**p < 0.01, *n* = 3) and Runx2 (**p < 0.01, *n* = 3) were significantly higher in the BCP/osteogenic-group MSCs than in the coralline-derived-HA/osteogenic-group MSCs

Figure 3 presents the Western blotting results for the expression of osteogenic proteins in the MSCs. Compared to a control group, the expression of the detected osteogenic protein in the coralline-HA-group MSCs and in the BCP-group MSCs was considerably enhanced. After 14 days of culture, the protein levels of leptin-R (BC14/BO14 0.12 ± 0.03-fold, **p < 0.01, *n* = 3), leptin (BC14/BO14 0.70 ± 0.06-fold, *p < 0.05, *n* = 3), and Runx2 (BC14/BO14 0.65 ± 0.07-fold, *p < 0.05, *n* = 3) were significantly higher in the BCP/osteogenic-group MSCs than in the BCP/control-group MSCs. Additionally, the protein levels of leptin-R (HO14/BO14 0.24 ± 0.04-fold, **p < 0.01, *n* = 3) and Runx2 (HO14/BO14 0.61 ± 0.04-fold, **p < 0.01, *n* = 3) were significantly higher in the BCP/osteogenic-group MSCs than in the coralline-HA/osteogenic-group MSCs.

#### Quantitative measurement of ALP

**Fig. 4:**
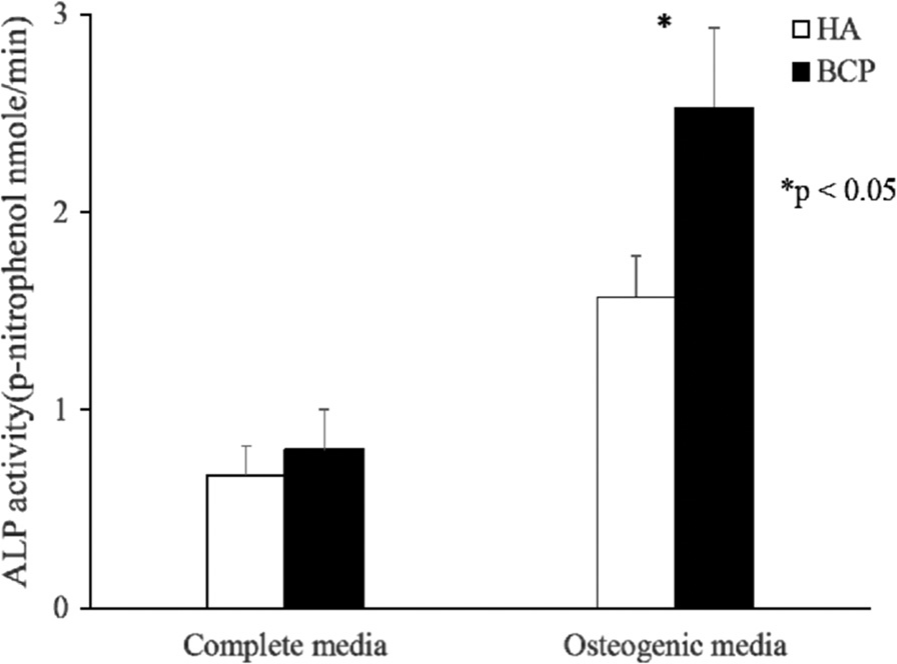
Quantitative measurement of ALP. MSCs cultured in PLGA/coralline-derived HA hybrid scaffolds or PLGA/BCP hybrid scaffolds had significantly lower ALP activity levels in the control (complete) medium than in the osteogenic medium throughout the 14-day culture period. In addition, ALP activity levels in the PLGA/BCP-group MSCs cultured in the osteogenic medium were significantly higher than those in the PLGA/coralline-derived-HA-group MSCs throughout the 14-day culture period (*p < 0.05, *n* = 3)

Figure 4 presents the results of ALP activity measurements. MSCs cultured in a PLGA/coralline HA hybrid scaffold or PLGA/BCP hybrid scaffold had significantly lower ALP activity levels in the control (complete) medium than in the osteogenic medium throughout the 14-day culture period. In addition, ALP activity levels in the PLGA/BCP-group MSCs cultured in osteogenic medium were significantly higher than those in the PLGA/coralline-HA-group MSCs throughout the 14-day culture period (PLGA/coralline HA vs. PLGA/BCP 1.57 ± 0.21 vs. 2.53 ± 0.4, *p* < 0.05, *n* = 3).

### In vivo studies

#### Computed tomography findings

**Fig. 5:**
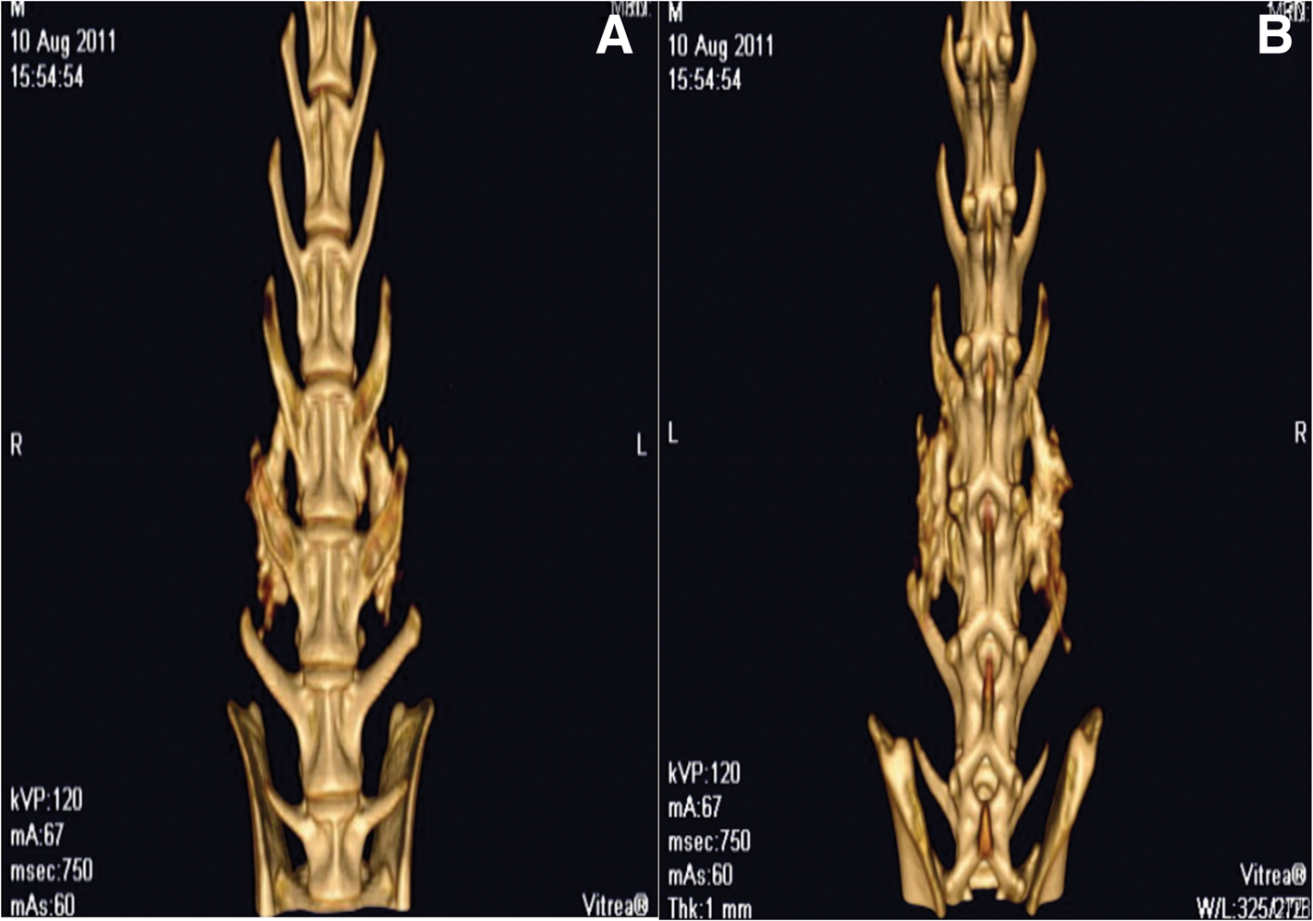
Computed tomography findings. Three-dimensional CT reconstructions revealed the presence of continuous bone bridges and satisfactory fusion mass incorporated with the transverse processes (**a**, **b**)

3D CT reconstructions revealed the presence of continuous bone bridges and satisfactory fusion mass incorporated with the transverse processes (Fig. 5a, b).

#### Dual-energy X-ray absorptiometry examination

**Fig. 6:**
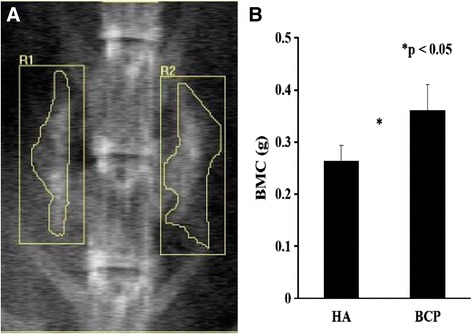
Dual-energy X-ray absorptiometry examination. Radiographs of the specimens retrieved from the BCP graft and HA graft showed continuous trabecular bone patterns in the area between the transverse processes (**a**). The BMC values were higher in the BCP graft than in the coralline-derived HA graft (**b**; *p < 0.05, *n* = 3)

Radiographs of the specimens retrieved from the BCP graft and coralline HA graft showed continuous trabecular bone patterns in the area between the transverse processes (Fig. 6a). The BMC values were higher in the BCP graft than in the coralline HA graft (Fig. 6b; BCP vs. coralline HA 0.36 ± 0.05 vs. 0.26 ± 0.03 g, *p < 0.05, *n* = 3).

## Discussion

A recent study proposed the theory that certain materials could trigger the secretion of factors leading to bone formation rather than simply causing the accumulation of such factors [27]. Other studies have focused on the direct effects of different materials on various types of multipotential cells that can eventually differentiate into osteocytes, such as adipose-derived stem cells [28], C2C12 cells [29], and bone marrow MSCs [30]. The MSCs used here were characterized by the presence of a consistent set of marker proteins on their surface. The percentages of cells expressing the human MSC markers CD105, Stro-1, and α-SMA are shown in Fig. 1.

Up to now, a series of biomaterials have been reported to be osteoinductive, including demineralized bone matrix [16], porous HA ceramics [31], and TCP ceramics [3]. Although these findings expanded the understanding of osteoinductive biomaterials, the osteoinductive mechanism of those biomaterials behind the phenomenon of ectopic formation is still not fully understood. Tan et al. observed that HA and BCP induced the expression of osteogenic markers in C2C12 cells, but they did not quantify the differences between the two ceramics [29]. Barradas et al. showed that MSCs cultured in β-TCP express more OPN, OC, collagen type I (Col I), and bone sialoprotein (BSP) than MSCs cultured in HA after 7 days [32]. In the present study, coralline HA hybrid scaffold and BCP hybrid scaffold were also found to upregulate the protein expression of osteogenic markers of MSCs in the osteogenic medium (Fig. 3). In addition, we found higher mRNA expression of OPN, Runx2, and OSC (Fig. 2); higher protein levels of leptin, leptin-R, and Runx2 (Fig. 3); and higher ALP activity (Fig. 4) in MSCs grown in the PLGA/BCP hybrid scaffold which were cultured in the osteogenic medium. All of the above findings make it more reasonable to conclude that the osteoinductive feature of these materials might rely on their ability to directly induce the differentiation of MSCs along an osteoblastic lineage.

Several signaling pathways and molecules have been shown to be critical for MSC differentiation along an osteogenic lineage [33, 34]. To explore the critical molecules initiating the osteoblastic differentiation of cells stimulated by calcium phosphate materials, Tang et al. proved that the osteoinductive calcium phosphates, both HA and BCP, could upregulate the gene expression of BMPs, in addition to even upregulating the expression of intracellular signal transduction molecules in the Smad pathway during osteogenesis in MSCs [35]. Leptin exerts its effect by binding to leptin-R and activating the JAK/STAT pathway, which is highly expressed in human osteoblasts [36, 37]. In the present study, the BCP hybrid scaffold culture resulted in higher upregulation of mRNA (Fig. 2) and protein (Fig. 3) expression of leptin-R in MSCs than did the coralline HA hybrid scaffold. Leptin [19] and leptin-R [20] are expressed in MSCs and might act as a local (autocrine) factor in the osteogenesis of MSCs and bone remodeling. Enhancement of the expression of transforming growth factor (TGF)-β [38] and ALP [39] by leptin has previously been reported, and the stimulatory effect of leptin on the osteogenesis of MSCs was also confirmed by the ALP activity measured in this study (Fig. 4).

Resorbable devices have been used in applications such as fracture fixation, bone grafting, spinal fusion, and deformity correction. A previous study showed the HA-grafted implants were poorly anchored compared with allografted implants, suggesting that coralline HA granules should be considered a bone graft extender rather than a bone graft substitute [40]. Coralline-derived HA typically performs in an inferior manner to other HA materials in implant fixation. However, coralline-derived HA representing as a promising bone graft extender in spinal fusion has also been reported [41]. In addition, a system review showed the overall fusion rate for all ceramic products (including β-TCP, BCP, calcium sulfate, coralline hydroxyapatite, synthetic hydroxyapatite, silicated hydroxyapatite) as a bone graft extender in the lumbar spine was 86.4 % in 30 studies with 1332 patients. The data for coralline hydroxyapatite (Pro-Osteon 200, Pro-Osteon 500) was 86.9 % in 7 studies with 168 patients. The coralline-derived HA typically performs in a similar manner to other HA materials in the lumbar spinal fusion [42]. In our previous study, we showed that the MSC/coralline-derived HA/type I collagen hybrid graft could be effectively used to achieve posterolateral spinal fusion [9]. In this study, we further investigated the gene, mRNA, and protein expression of MSCs cultured on BCP hybrid scaffolds and coralline-derived HA hybrid scaffolds (Figs. 2, 3, and 4). In addition, we put these MSC–BCP hybrid scaffolds and MSC–coralline-derived HA hybrid scaffolds into rabbits for spinal fusion test (Figs. 5 and 6).

Previous studies demonstrated that HA/TCP stimulated the osteogenic differentiation of MSCs [43, 44]. In this study, BCP hybrid scaffolds were performed using MASTERGRAFT, a commercial composite consisting of 15 % HA and 85 % β-TCP. HA is a material with biomechanical properties similar to natural bone. Briefly, when compared to β-TCP, HA is a more stable phase under physiological conditions, as it has a lower solubility and, thus, slower resorption kinetics [45, 46]. Therefore, the BCP concept is determined by the optimum balance of a more stable phase of HA and a more soluble TCP. Due to a higher biodegradability of the β-TCP component, the reactivity of BCP increases with the TCP/HA ratio increasing.

Differences in the porosity and surface area of particles within the material can influence its performance characteristics. Porosity is defined as a percentage of voids in solids and this morphological property is independent of the material. The surface area of porous bodies is much higher, which guarantees a good mechanical fixation in addition to providing sites on the surface that allow chemical bonding between the bioceramics and bones [47]. In addition, pore dimensions are also important. The dimensions of open pores are directly related to bone formation, since such pores grant both the surface and space for cell adhesion and bone ingrowth [48]. In this study, coralline-derived HA hybrid scaffolds were performed using the commercial product Pro-Osteon 500. Pro-Osteon 500 is prepared by the hydrothermal conversion (260 °C, 15,000 psi) of coral (consisting of mostly CaCO_3_, calcite form) in the presence of ammonium phosphate to hydroxyapatite [49]. Pro-Osteon 500 has a porosity rate of 60–70 % and a mean pore size of 500 μm. BCP hybrid scaffolds were performed using MASTERGRAFT. BCP of varying HA to β-TCP weight ratios is obtained by sintering precipitated calcium-deficient apatite (Ca/P molar ratio <1.67). MASTERGRAFT has a porosity rate of 80 % and a mean pore size of 500 μm. The mean pore size between the two calcium phosphate materials is equal, but the porosity rate is higher in MASTERGRAFT than in Pro-Osteon 500. This may be one of the reasons why the BMC values were higher in BCP hybrid scaffolds than in coralline-HA hybrid scaffolds (Fig. 6).

To analyze the osteoinductive potential of ceramics in vivo, a previous study conducted ectopic implantation in the muscle tissue and femoral cortical bones of dogs [50]. In muscle, histomorphometric analysis showed earlier and more bone formed in BCP than in HA. In femoral cortical bone defects, significantly more bone was formed in BCP than in HA. Similarly, a comparison between HA and BCP sintered at the same temperature (thus with similar macro- and microstructural features but different chemistry) showed a more pronounced bone formation in BCP [51]. Our previous study showed that an MSC/coralline HA/type I collagen hybrid graft could be effectively used to achieve posterolateral spinal fusion in a rabbit model [9]. In the present study, we used the clinically relevant posterolateral spinal fusion model in which two materials can be compared in a paired manner. In our results, 3D reconstructed CT images showed continuous bone bridges and fusion mass incorporated with the transverse processes (Fig. 5). BMC values were higher in BCP hybrid scaffolds than in coralline HA hybrid scaffolds (Fig. 6). These results indicated that the osteogenic capacities of osteoinductive materials in osseous fusion sites are correlated: the higher the osteoinductive potential of the material, the faster the formation of the bone bridges.

To provide more information, we show the microarray analysis of MSCs cultured on coralline-derived HA hybrid scaffolds and BCP hybrid scaffolds in Table 1. Higher levels of osteogenic marker expression in BCP hybrid scaffolds were shown (Runx2 and leptin-R). In addition, higher levels of MAFB and IQGAP1 and lower levels of COMP expression in BCP hybrid scaffolds were shown. MAFB (v-maf musculoaponeurotic fibrosarcoma oncogene homolog B) is a biomarker for rheumatoid arthritis [52]. IQGAP1 (IQ motif containing GTPase activating protein 1) modulates many different signaling pathways and cellular functions, including mitogen-activated protein kinase (MAPK) signaling, Ca^2+^/calmodulin signaling, cell–cell adhesion, β-catenin-mediated transcription, and microbial invasion [53]. COMP (cartilage oligomeric matrix protein) is a biomarker for cartilage degradation [54]. However, the role of MAFB, IQGAP1, and COMP in osteogenesis of MSCs needs to be further investigated.

In conclusion, the results of the present study indicate that a BCP culture is more effective than an HA culture at inducing the osteogenesis of MSCs due to the greater capacity of the BCP culture to upregulate the expression of leptin receptors and even the intracellular signal transduction molecules of osteogenesis of MSCs. This is correlated to in vivo data, where BCP induced more bone formation in a rabbit spinal fusion model.

## Abbreviations

MSCs: Mesenchymal stem cells
HA: Hydroxyapatite
BCP: Biphasic calcium phosphate
PLGA: Poly(lactide-*co*-glycolide)
ALP: Alkaline phosphatase
Q-PCR: Quantitative polymerase chain reaction
CT: Computed tomography
DEXA: Dual-energy X-ray absorptiometry
Leptin-R: Leptin receptor
BMC: Bone mineral content
HSC: Hematopoietic stem cell
BSP: Bone sialoprotein
MAFB: V-maf musculoaponeurotic fibrosarcoma oncogene homolog B
IQGAP1: IQ motif containing GTPase activating protein 1
COMP: Cartilage oligomeric matrix protein, biomarker for cartilage degradation
